# Fecal incontinence as the sole presentation of focal epilepsy; a case report

**DOI:** 10.1093/omcr/omad064

**Published:** 2023-06-26

**Authors:** Laith T Al-Ameri, Ekhlas K Hameed, Bilal S Maroof, Hayder Al-Momen

**Affiliations:** Al-Kindy College of Medicine, University of Baghdad, Baghdad, Iraq; Al-Kindy College of Medicine, University of Baghdad, Baghdad, Iraq; Al-Kindy College of Medicine, University of Baghdad, Baghdad, Iraq; Al-Kindy College of Medicine, University of Baghdad, Baghdad, Iraq

## Abstract

Focal epilepsy is a surge in brain activity arising from a localised area of the cerebral cortex; it can be sub-classified in different categories including motor, sensory, autonomic and cognitive subtypes. A clinical case report of a 11-year-old girl was diagnosed with frequent fecal incontinence four or more times daily for more than two months. An electroencephalogram (EEG) study suggested a prominent interictal spike and sharp wave discharge on the left hemisphere, mainly at the frontotemporal region without loss of consciousness or even speech disruption. This could be due to the normal EEG study of the dominant hemisphere. A magnetic resonance imaging study was done to exclude space-occupying lesions or focal lesions of the left hemisphere of the brain. An impression was made with abnormal EEG showing focal epileptiform activity as a final diagnosis. The patient was treated with Leviteracetam anti-epileptic drug 250 mg twice daily with significant clinical improvement at a 3-month follow-up.

## INTRODUCTION

Recently, vast attention was brought toward epilepsy with an explosion of information regarding etiology, pathophysiology, and different behavioral, medical and surgical management approaches. Epilepsy is a common condition seen very frequently in neurological practice having a great impact on health with variable cognitive, psychiatric and functional comorbidities with a negative social impact [1, 2].

Among 1% of the population diagnosed with epilepsy during their life, 75% are of the pediatric age group, with focal epilepsy being the most common subtype [3].

As a major type, focal epilepsy could be defined as a surge in brain activity arising from a localised area of the cerebral cortex. Variable signs and symptoms were reported depending mainly on the area of brain activity. However, according to symptoms variety, focal epilepsy may be sub-classified in different categories including motor, sensory, autonomic, and cognitive subtypes. Additionally, many focal epileptic patients experienced a warning sign (aura), such as the loss of awareness, strange smell, etc. [4].

## CASE REPORT

A 11-year-old, right-handed girl weighted 36 kg and no previous history of epilepsy or febrile convulsion. She was born by normal vaginal delivery after a full-term pregnancy with no remarkable medical conditions such as brain infection, brain trauma or any diagnosed metabolic disease. No previous history of convulsions was recorded. However, the patient was diagnosed by her family as having frequent fecal incontinence four or more times daily for more than 2 months. No other symptoms, triggers or warning signs were noted.

The following possible differential diagnoses were excluded through proper medical history, examination and investigations: chronic constipation, cerebral palsy, neurogenic bowel, Hirschsprung disease, coeliac disease, anorectal malformation, spina bifida, spinal dysraphism, myelomeningocele, neurogenic bowel and tethered spinal cord.

The abdominal ultrasound, abdominal X-ray with contrast, bowel motility study, rectal wall biopsy, Coeliac immunological screen, brain and spinal magnetic resonance imaging all showed normal results.

The whole workup re-evaluation was done aiming to find out the diagnosis for this presentation. A breakthrough piece of information in the medical history caught the attention of the treating physicians when the family told them about a family history of epilepsy. In our community, such a disease could be considered as social stigmata and people are trying to avoid discussing these issues. Then, considering her family history of epilepsy, an electroencephalogram (EEG) was ordered and performed.

**Figure 1:**
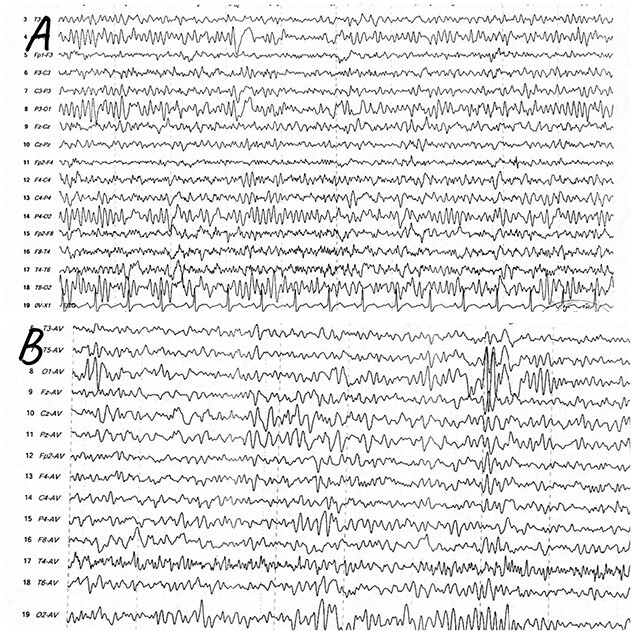
(**A**) EEG (bipolar montage) with left frontotemporal periodic lateralized epileptiform discharge. (**B**) Multifocal epileptiform discharge (average montage) most prominent over the left frontotemporal region.

While the patient was conscious, alert and cooperative, an EEG examination (with Cadwell ARC essentia device brand) was performed through 18 channels in a bipolar mode of activity with a microvolt sensitivity of 10. A 20-min waking period was obtained, including opening and closing of the eyes, with 3 min of hyperventilation and intermittent photic stimulation at 10, 20 and 30 frequencies as an evocative procedure. Results were suggested as a well-organised background activity in the range of complex partial seizures (CPS), responses to eye-opening and closing were normal, hyperventilation causes slowing of clear asymmetry and transients of fronto-temporal sharp were noted; the detailed EEG information shown in Figure 1. A magnetic resonance imaging (MRI) study was done to exclude space-occupying lesions or focal lesions of the right hemisphere of the brain. An impression was made with abnormal EEG showing focal epileptiform activity as a final diagnosis. The patient was treated with Leviteracetam anti-epileptic drug 250 mg twice daily with significant clinical improvement at a 3-month follow-up with no potential side effects of prescribed anti-epileptic medication reported.

## DISCUSSION

Studies showed that some seizure types could be followed by many unusual symptoms such as fecal incontinence with some degree of unconsciousness, like Benign Rolandic Epilepsy. Regarding this case, the patient underwent fecal incontinence without a loss of consciousness with a prominent interictal spike and sharp wave discharge on the left hemisphere, mainly at the frontotemporal region on EEG study without a loss of consciousness or even speech disruption. This could be due to fact that most of insular area in human is found on the left hemisphere with a high connection with the prefrontal area, so ictal activities or lesion effects in this area may cause painless distended rectum and defecation to occur accidentally with totally intact consciousness [5]. Several cases were reported as fecal incontinence or an urge to defecate, but with the presence of intermittent fall as the main manifestation. A previously published short communication reported two children with idiopathic partial epilepsy associated with fecal incontinence, pointing out to the role of alteration in the cortical area involved in the regulation of muscular tone of pelvic floor muscles through nearby epileptic activity [6]. Another case report has discussed a middle-aged female with partial epilepsy and an urge to defecate associated with visual symptoms and a secondary generalized tonic–clonic fit, suggesting a cortical lateralization [7]. However, in our reported case, normal neurological examination and brain MRI with abnormal focal epileptiform discharge on the right frontotemporal region confined the diagnosis to idiopathic focal epilepsy that led to fecal incontinence and a significant clinical improvement following antiepileptic treatment after a 1-month follow-up. Other studies with a specific emphasis on pathophysiology are required to confirm and explain rare symptoms.

## CONFLICT OF INTEREST STATEMENT

None declared.

## FUNDING

Authors declare no funding to be reported.

## ETHICAL APPROVAL

Not required.

## INFORMED CONSENT

A written consent was obtained from patient’s parents for research and publication.

## GUARANTOR

Laith Al-Ameri.
